# Evaluation of the smear layer removal and erosive capacity of EDTA, boric acid, citric acid and desy clean solutions: an in vitro study

**DOI:** 10.1186/s12903-015-0090-y

**Published:** 2015-09-03

**Authors:** Tugba Turk, Mehmet Emin Kaval, Bilge Hakan Şen

**Affiliations:** Department of Endodontology, Ege University, School of Dentistry, 35100 Izmir, Turkey; Private Practise, İzmir, Turkey

## Abstract

**Background:**

The purpose of this study was to investigate the smear layer removal and erosive capacity of various irrigation solutions with sequential use of NaOCl on instrumented root canal walls.

**Methods:**

The root canals of single-rooted teeth were instrumented with ProTaper rotary instrument. Then, the teeth were randomly divided into five experimental groups. The root canals were irrigated with one of the following solutions (5 mL/1 min): 5 % EDTA, 5 % boric acid (BA), a mixture of BA and CA, 2.5 % citric acid (CA) and 5 % Desy Clean. After irrigating with 2.5 % NaOCl and distilled water, the roots were split into two halves and each half was prepared for SEM examination. Representative photographs were taken from each third at x500 and x1000 magnifications. Double blind scoring was performed by two calibrated observers for smear layer and erosion. The scores were statistically analyzed using Kruskal-Wallis, Dunn’s *post hoc* and Spearman’s correlation tests (*p* = 0.05).

**Results:**

There were statistically significant differences among the solutions by means of smear layer and erosion (*p* < 0.05). While 2.5 % CA solution was the most effective solution in removal of smear layer, it was also the most erosive solution (*p* < 0.05). 5 % Desy Clean removed smear layer effectively and caused less erosion. There was a negative, but statistically significant correlation between presence of smear layer and erosion (*r* = −0.684; *p* < 0.0001).

**Conclusion:**

Desy Clean can be a promising agent as an irrigation solution with optimal smear layer removal capacity and less erosive effects.

## Background

During endodontic treatment, root canal instrumentation produces a smear layer, which consists of organic and inorganic materials. This layer covers the instrumented walls and may prevent the penetration of intracanal medicaments into the dentinal tubules and may negatively affect the adaptation of the root canal filling materials to the root canal walls [1]. Currently, the most common method for smear layer removal is the sequential use of ethylene diamine tetraacetic acid (EDTA) and sodium hypochlorite (NaOCl) solutions [2, 3]. Additionally, citric acid solution is another solution choice for removal of smear layer and the clinical efficiency of this organic acid solution has been reported previously [4].

Even though EDTA and citric acid solutions are effective for removal of smear layer, both solutions cause erosion of peritubular and intertubular dentin and reduce the dentin microhardness [5]. In addition, NaOCl irrigation following EDTA increases dentinal erosion effect [6]. Because of these adverse effects of acidic irrigation solutions, studies are focused on new irrigation solutions [7, 8]. However, an ideal solution which can remove smear layer effectively without causing erosion on root canal dentin walls have not been found yet.

Desy Clean (Sojall, Salzburg, Austria) solution contains sorbic acid (0.15 ml/L), hydrogen peroxide (128 ml/L), sodium benzoate (0.21 ml/L), acetic acid (26.64 ml/L) and water (845 ml/L). The manufacturer claims that 5 % Desy Clean possesses promising antibacterial activity and high biocompatibility (http://desyclean.com/desy-clean-ekolojik-dezenfektan-katalog.pdf). Boric acid has antiseptic, anti-bacterial and anti-fungal properties [9]. It has been used in medicine for the treatment of Otitis externa, for elimination of recurrent vaginal yeast infections and for dressing minor burns and cuts [10]. Both solutions have not been tested yet in the complex root canal system and their efficiency in removal of the smear layer is uncertain.

Therefore, the purpose of this study was to investigate the smear layer removal and erosive capacity of 5 % EDTA, 5 % boric acid (BA), 2.5 % citric acid (CA), a combination of 5 % BA and 2.5 % CA, and 5 % Desy Clean solutions with sequential use of NaOCl on instrumented root canal walls.

## Materials and Methods

This study was approved by the Medical Ethics Committee of Ege University, in accordance with the Declaration of Helsinki (Reference number: 1411111). Twenty-five intact single and straight (less than 5° curvature) rooted teeth extracted for periodontal or prosthetic reasons were collected for this study. The teeth were then stored in 0.1 % thymol solution at 4 °C until use. After the crowns were removed at the cement-enamel junction, the working length was determined with a #10 K-file. The file was inserted into the canal until it was seen at the apical foramen. Then 1 mm was subtracted from this length to establish the working length.

**Fig. 1:**
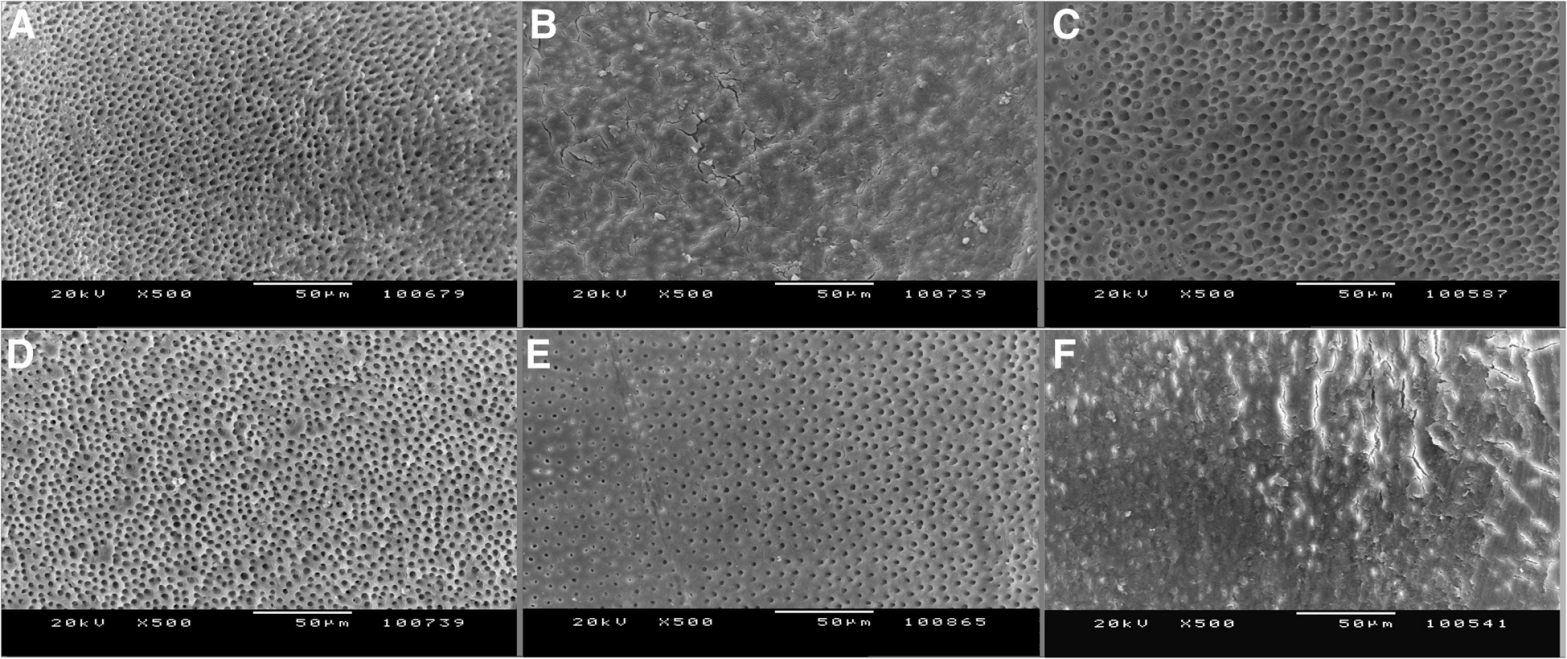
Representative photomicrographs for smear layer (original magnification x500). **a** 5 % EDTA, **b** 5 % BA, **c** 2.5 % CA, **d** 2.5 % BA and 2.5 % CA, **e** Desy Clean, **f** Control group

**Fig. 2:**
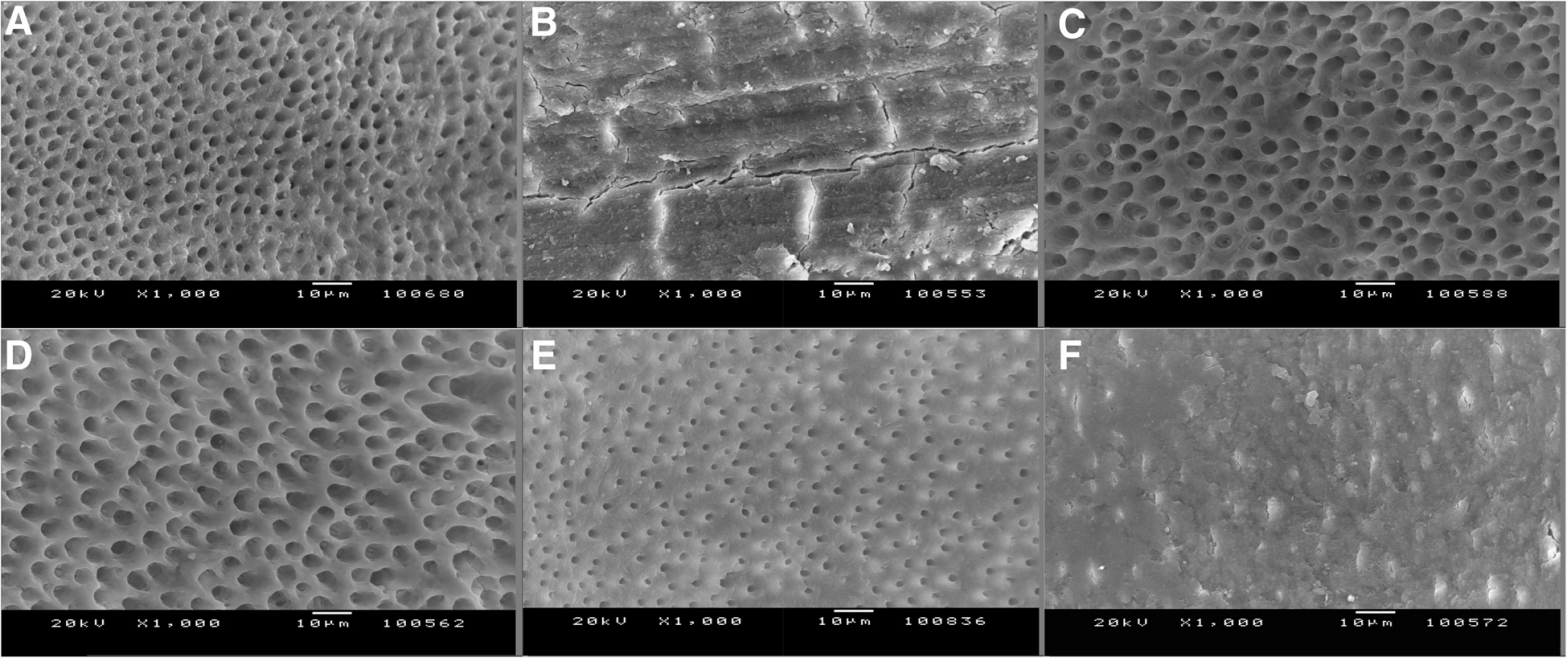
Representative photomicrographs for erosion (original magnification x1000). **a** 5 % EDTA, **b** 5 % BA, **c** 2.5 % CA, **d** 5 % BA and 2.5 % CA, **e** Desy Clean, **f** Control group

All teeth had their apices sealed with wax to prevent the outward flow and to stimulate in vivo apical counter pressure during canal preparation. Specimens were instrumented with ProTaper rotary instruments (Dentsply Maillefer, Ballaigues, Switzerland) up to size F5. After each instrument, 1 mL 2.5 % NaOCl was used for irrigation with a 27-gauge irrigation needles (KerrHawe, SA, Bioggio, Switzerland) attached to a 2-mL syringe. After instrumentation, the teeth were divided randomly into five experimental groups according to final irrigating solution: 5 % EDTA (group 1), 5 % BA (group 2), a mixture of 5 % BA and 2.5 % CA (group 3), 2.5 % CA (group 4), and 5 % Desy Clean (group 5). Then, each root canal was irrigated with 2.5 % NaOCl and distilled water. During the final irrigation procedure 27-gauge needles were attached to a 5-mL syringe, and were positioned 1 mm short of working length. Irrigation time for every solution was 1 min. As control, five specimens were prepared same as the test groups, but not irrigated with the acids or chelators, and the presence of an adequate smear layer and un-eroded dentine before final irrigation was confirmed (Figs. 1f and 2f).

The roots were split into two halves and each half was prepared for SEM examination (Jeol JSM-5200 Tokyo, Japan). Thus, 10 specimens were obtained for each group. All samples were blinded before SEM evaluation. Three photographs were taken from randomly selected areas of each third (apical, middle and coronal) both for ×500 and ×1000 magnifications. A total 900 photographs were evaluated in a blind manner by two observers at two different sessions. Separate evaluations were done for smear layer (×500) and erosion (×1000). The average of the observers’ scorings for each section was used for the statistical analysis.

Smear layer was evaluated according to a numeric evaluation scale [11]: 1, no smear layer, dentinal tubules open; 2, small amount of smear layer, some dentinal tubules open; 3, homogeneous smear layer covering the root canal wall, few dentinal tubules open; 4, complete root canal wall covered by a homogeneous smear layer, no open dentinal tubules; 5, heavy nonhomogeneous smear layer covering the complete root canal wall.

In addition, the degree of erosion of the dentinal tubules was scored according to a classification by Torabinejad et al. [7]: 1, no erosion, all tubules in normal appearance and size; 2, moderate erosion, the peritubular dentin was eroded; 3, severe erosion, the intertubular dentin was destroyed and tubules were connected with each other. The scores were statistically analyzed using Kruskal-Wallis, Dunn’s *post hoc* and Spearman’s correlation tests (*p* = 0.05). Kappa test was used to test intra-examiner agreement and inter-examiner agreement.

## Results

The Kappa test for both smear and erosion observations showed high (good) intra-examiner agreement and inter-examiner agreement values ranging from 0.61 to 0.78. (*For erosion*: 1. Intra-examiner agreement value 0.75; 2. Intra-examiner agreement value 0.61; inter-examiner agreement value 0.64. *For smear layer*: 1. Intra-examiner agreement value 0.68; 2. Intra-examiner agreement 0.66; inter-examiner agreement value 0.78).

Figures 1 and 2 shows representative images of each group (×500 for smear layer and ×1000 for erosion).

### Smear layer

The smear layer scores for each group are presented in Table 1. When the different thirds were compared for the effectiveness of solutions on removal of smear layer, all solutions were more effective in the coronal third, but the differences were not significant (*p* > 0.05). On the other hand, there were statistically significant differences among the effect of the solutions (*p* < 0.05). While 2.5 % CA solution was the most effective solution in removal of smear layer (*p* < 0.05), 5 % BA received the highest scores (*p* < 0.05). There were no significant differences between 5 % Desy Clean and CA groups by means of smear layer removal in any region (*p* > 0.05).

**Table 1:** Mean smear layer scores for each group and each region

Groups	Coronal	Middle	Apical
1 (5 % EDTA)	1.94^a^	2.07^a^	2.25^a^
2 (5 % BA)	3.95^b^	3.97^b^	4.41^b^
3 (5 % BA & 2.5 % CA)	1.49^e^	1.91	2.46^c^
4 (2.5 % CA)	1.33	1.36	1.33^d^
5 (5 % Desy Clean)	1.78	1.65	1.95

^a^Significant difference between groups compared to group 2 and 4

^b^Significant difference between groups compared to group 3, 4 and 5

^c^Significant difference between groups compared to group 4

^d^Significant difference between groups compared to group 5

^e^Significant difference within group locations compared to apical third

Same superscript letter in a row/colum are significantly different (*p* > 0.05)

### Erosion

The erosion scores for each group are listed in Table 2. There was no significant difference among all thirds by means of erosion (*p* > 0.05). However, there were statistically significant differences among the solutions (*p* < 0.05). While 5 % boric acid had the lowest erosion scores, 2.5 % CA received the highest erosion scores (*p* < 0.05).

**Table 2:** Mean erosion scores for each group and each region

Groups	Coronal	Middle	Apical
1 (5 % EDTA)	1.37^a^	1.10^b^	1.03^a^
2 (5 % BA)	1.03^a^	1.00^a^	1.00^a^
3 (5 % BA & 2.5 % CA)	2.42^c^	2.25^c^	1.70^d,e^
4 (2.5 % CA)	2.67^c^	2.36^c^	2.27^c^
5 (5 % Desy Clean)	1.15	1.14	1.10

^a^Significant difference between groups compared to group 3 and 4

^b^Significant difference between groups compared to group 2,3 and 4

^c^Significant difference between groups compared to group 5

^d^Significant difference between groups compared to group 4 and 5

^e^Significant difference within group locations compared to apical coronal and middle third

There was a negative, but statistically significant correlation between presence of smear layer and erosion (*r* = −0.684; *p* < 0.0001).

## Discussion

Review of the literature reveals that smear layer removal has been considered as an important step during root canal treatment [12, 13]. A variety of chemicals with a broad range of concentrations and different irrigation regimes have been used to remove this layer [1, 14]. However, irrigants and delivery systems cause alterations in the chemical and structural composition of dentin during removal of smear layer [15–19]. Recently, Uzunoğlu et al. [20] stated that fracture resistances of root canal-treated teeth were affected by irrigation procedures. Ideally, mechanical properties like strength, composition and hardness of dentin should not be affected in any negative aspect after irrigation procedures or this effect should be minimized. However, the sequential use of EDTA (or any acid) and NaOCl causes a progressive dissolution of dentin at the expense of peritubular and intertubular areas [21].

In this ex vivo study, boric acid did not remove smear layer, while, in contrast, citric acid, EDTA and Desy Clean removed this layer effectively at all thirds. On the other hand, EDTA and citric acid caused significantly more erosion, while Desy Clean showed minimal erosive effect. Erosion findings for EDTA and citric acid are in agreement with previous studies [18, 19, 22]. According to Reis et al. [23], concentration of citric acid has significant influence on its chelating ability. Lower concentrations of acidic solutions have been recommended for root canal treatment to avoid undesirable erosion of root canal dentin [18, 19, 24]. In the present study, a lower concentration of citric acid (2.5 %) was used. Even at this lower concentration, it caused considerable erosion. Sen et al. [19] investigated the smear layer removal and erosive capacities of different concentrations of EDTA (15 %, 10 %, 5 %, and 1 %) on instrumented root canal walls and concluded that EDTA concentration could be as low as 1 % for clinical use, because it still removed smear layer adequately and caused less dentinal erosion.

Teixeria et al. [25] reported that 1, 3 and 5 min application times for EDTA and NaOCl were equally effective for removal of the smear layer; additionally Saito et al. [26] reported that 1 min application time of EDTA and NaOCl solutions is efficient in removing smear layer on the root canal walls. Sen et al. [19] preferred also 1 min application time for each final irrigation solution and their results were in agreement with Saito et al. [26]; therefore in the present study contact time of each irrigant was set at 1 min during the final irrigation procedure.

Boron, which is abundant as boric acid and borate, has anti-inflammatory effects by regulating oxidant-antioxidant levels of tissue [27, 28]. Luan et al. [29] showed that topical use of boron is effective in treating periodontal disease. In addition, boric acid has considerable antimicrobial effects [30]. Because smear layer removal capacity of this solution is insufficient, a combination of boric and citric acid solutions can be mixed for clinical use in order to achieve antimicrobial effect and smear layer removal capacity at the same time.

One of the most important finding in the present study was that 5 % Desy Clean had less erosive effects while removing the smear layer. 5 % Desy Clean’s pH is 2.5–3.5 (http://desyclean.com/desy-clean-ekolojik-dezenfektan-katalog.pdf); therefore, its acidic nature helps removing the smear layer, but it does not cause erosion at the present concentration and application time. It can react easily with macromolecules such as membrane lipids and DNA; hence, resulting in bacterial death [31].

## Conclusion

Desy Clean is an environmental friendly sterilizing agent without chlorine, formaldehyde and alcohol and this solution can be a promising agent as an irrigation solution with optimal smear layer removal capacity and less erosive effects.

## Abbreviations

EDTA: Ethylene diamine tetraacetic acid
BA: Boric Acid
CA: Citric Acid
NaOCl: Sodium hypochlorite
SEM: Scanning electron microscope
mL: Mililiter

## References

[1] Violich DR, Chandler NP (2010). Smear layer in endodontics – a review. Int Endod J.

[2] Abbott PV, Heijkoop PS, Cardaci SC, Hume WR, Heithersay GS (1991). An SEM study of the effects of different irrigation sequences and ultrasonics. Int Endod J.

[3] Cruz-Filho AM, Sousa-Neto MD, Savioli RN, Silva RG, Vansan LP, Pécora JD (2011). Effect of chelating solutions on the microhardness of root canal lumen dentin. J Endod.

[4] Pérez-Heredia M, Ferrer-Luque CM, González-Rodríguez MP (2006). The effectiveness of different acid irrigating solutions in root canal cleaning after hand and rotary instrumentation. J Endod.

[5] Qian W, Shen Y, Haapasalo M (2011). Quantitative analysis of the effect of irrigant solution sequences on dentin erosion. J Endod.

[6] Niu W, Yoshioka T, Kobayashi C, Suda H (2002). A scanning electron microscopic study of dentinal erosion by final irrigation with EDTA and NaOCl solutions. Int Endod J.

[7] Torabinejad M, Khademi AA, Babagoli J, Cho Y, Johnson WB, Bozhilov K, Kim J, Shabahang S (2003). A new solution for the removal of the smear layer. J Endod.

[8] da Silva LA, Sanguino AC, Rocha CT, Leonardo MR, Silva RA (2008). Scanning electron microscopic preliminary study of the efficacy of SmearClear and EDTA for smear layer removal after root canal instrumentation in permanent teeth. J Endod.

[9] Meers PD, Chow CK (1990). Bacteriostatic and bactericidal actions of boric acid against bacteria and fungi commonly found in urine. J Clin Pathol.

[10] del Palacio A, Cuétara MS, López-Suso MJ, Amor E, Garau M (2002). Randomized prospective comparative study: short-term treatment with ciclopiroxolamine (cream and solution) versus boric acid in the treatment of otomycosis. Mycoses.

[11] Hülsmann M, Rümmelin C, Schäfers F (1997). Root canal cleanliness after preparation with different endodontic handpieces and hand instruments: a comparative SEM investigation. J Endod.

[12] Kokkas AB, Boutsioukis A, Vassiliadis LP, Stavrianos CK (2004). The influence of the smear layer on dentinal tubule penetration depth by three different root canal sealers: an in vitro study. J Endod.

[13] Torabinejad M, Handysides R, Khademi AA, Bakland LK (2002). Clinical implications of the smear layer in endodontics: a review. Oral Surg Oral Med Oral Pathol Oral Radiol Endod.

[14] Gu LS, Kim JR, Ling J, Choi KK, Pashley DH, Tay FR (2009). Review of contemporary irrigant agitation techniques and devices. J Endod.

[15] Baumgartner JC, Ibay AC (1987). The chemical reactions of irrigants used for root canal debridement. J Endod.

[16] Marending M, Paqué F, Fischer J (2007). Zehnder M Impact of irrigant sequence on mechanical properties of human root dentin. J Endod.

[17] Zhang K, Tay FR, Kim YK, Mitchell JK, Kim JR, Carrilho M, Pashley DH, Ling JQ (2010). The effect of initial irrigation with two different sodium hypochlorite concentrations on the erosion of instrumented radicular dentin. Dent Mater.

[18] Çalt S, Serper A (2002). Time- dependent effects of EDTA on dentin structures. J Endod.

[19] Sen BH, Ertürk O, Pişkin B (2009). The effect of different concentrations of EDTA on instrumented root canal walls. Oral Surg Oral Med Oral Pathol Oral Radiol Endod.

[20] Uzunoglu E, Aktemur S, Uyanik MO, Durmaz V, Nagas E (2012). Effect of ethylenediaminetetraacetic acid on root fracture with respect to concentration at different time exposures. J Endod.

[21] Sim TP, Knowles JC, Ng YL, Shelton J, Gulabivala K (2001). Effect of sodium hypochlorite on mechanical properties of dentine and tooth surface strain. Int Endod J.

[22] Poggio C, Dagna A, Colombo M, Rizzardi F, Chiesa M, Scribante A (2012). Decalcifying effect of different ethylenediaminetetraacetic acid irrigating solutions and tetraclean on root canal dentin. J Endod.

[23] Reis C, De-Deus G, Leal F, Azevedo E, Coutinho-Filho T, Paciornik S (2008). Strong effect on dentin after the use of high concentrations of citric acid: an assessment with co-site optical microscopy and ESEM. Dent Mater.

[24] Calt S, Serper A (2000). Smear layer removal by EGTA. J Endod.

[25] Teixeira CS, Felippe MCS, Felippe WT (2005). The effect of application time of EDTA and NaOCl on intracanal smear layer removal: an SEM analysis. Int Endod J.

[26] Saito K, Webb TD, Imamura GM, Goodell GG (2008). Effect of shortened irrigation times with 17 % ethylene diamine tetra-acetic acid on smear layer removal after rotary canal instrumentation. J Endod.

[27] Demirer S, Kara MI, Erciyas K, Ozdemir H, Ozer H, Ay S (2012). Effects of boric acid on experimental periodontitis and alveolar bone loss in rats. Arch Oral Biol.

[28] Ince S, Kucukkurt I, Cigerci IH, Fatih Fidan A, Eryavuz A (2010). The effects of dietary boric acid and borax supplementation on lipid peroxidation, antioxidant activity, and DNA damage in rats. J Trace Elem Med Biol.

[29] Luan Q, Desta T, Chehab L, Sanders VJ, Plattner J, Graves DT (2008). Inhibition of experimental periodontitis by a topical boron-based antimicrobial. J Dent Res.

[30] Schmidt M, Schaumberg JZ, Steen CM, Boyer MP (2010). Boric acid disturbs cell wall synthesis in saccharomyces cerevisiae. Int J Microbiol.

[31] Block BB, Block SS (1991). Peroxygen compounds. Disinfection, Sterilization, and Preservation.

